# Cellular Mechanisms of Drosophila Heart Morphogenesis

**DOI:** 10.3390/jcdd2010002

**Published:** 2015-02-16

**Authors:** Georg Vogler, Rolf Bodmer

**Affiliations:** Development, Aging and Regeneration Program, Sanford-Burnham Medical Research Institute, 10901 N. Torrey Pines Road, La Jolla, CA 92037, USA; E-Mail: gvogler@sanfordburnham.org

**Keywords:** *Drosophila*, cardiogenesis, morphogenesis, tinman, Cdc42, congenital heart disease, non-muscle myosin, zipper

## Abstract

Many of the major discoveries in the fields of genetics and developmental biology have been made using the fruit fly, *Drosophila melanogaster.* With regard to heart development, the conserved network of core cardiac transcription factors that underlies cardiogenesis has been studied in great detail in the fly, and the importance of several signaling pathways that regulate heart morphogenesis, such as Slit/Robo, was first shown in the fly model. Recent technological advances have led to a large increase in the genomic data available from patients with congenital heart disease (CHD). This has highlighted a number of candidate genes and gene networks that are potentially involved in CHD. To validate genes and genetic interactions among candidate CHD-causing alleles and to better understand heart formation in general are major tasks. The specific limitations of the various cardiac model systems currently employed (mammalian and fish models) provide a niche for the fly model, despite its evolutionary distance to vertebrates and humans. Here, we review recent advances made using the *Drosophila* embryo that identify factors relevant for heart formation. These underline how this model organism still is invaluable for a better understanding of CHD.

## 1. Introduction

Congenital heart disease (CHD) is the most frequently diagnosed birth defect, with an incident rate of about 1% among newborns [1,2]. There are several genetic origins of CHD, such as copy-number variation (CNV), which affects gene dosage (as found in DiGeorge Syndrome and Williams–Beuren Syndrome), or mutations that affect a single gene, often a cardiac transcription factor (*i.e.*, NKX2.5, GATA4) or structural components of the contractile apparatus of the cardiomyocytes (such as MYH6; for a review, see [3,4]). However, the underlying genetics of the majority of cases of CHD (which are labeled “sporadic”) seems to be more complex and is likely caused by the interaction of independent alleles and loci (“polygenic”). While recent advances in sequencing technology have led to a drastic increase in the data available on genetic variants among patients’ genomes, it is still necessary to experimentally validate candidate genes that were computationally derived from such genomic data.

Understanding the biological basis of CHD requires a molecular understanding of the complex development of the heart itself, and despite the great efforts of the scientific community, our understanding of heart morphogenesis is still very incomplete. Current vertebrate models of heart development are mouse, chicken and zebrafish embryos (for reviews, see [5,6,7,8]), which are complemented by cell-based assays, including heart tissue engineered from induced pluripotent stem cells (iPSCs) [9]. These models established valuable concepts for the number and origin of cardiac progenitor cells, lineage restriction and the respective contributions of these cells to different regions of the heart. To study the complexity of the heart and heart morphogenesis, it is necessary to combine different approaches to complement the specific shortcomings of each model. This need has spurred the introduction and adoption of other models, such as tunicates [10], which are basic chordates, and even invertebrate model organisms, such as the fruit fly, *Drosophila melanogaster* [11,12,13,14].

In the last three decades, the powerful genetic model system, *Drosophila*, has been instrumental in the identification and dissection of the genetics of many developmental processes, including heart development (for a review, see [15]), serving as a pioneer for higher model organisms. *Drosophila* is an extremely versatile model, with a plethora of reagents and genetic tools available, including RNAi lines to tissue-specific targeting of each fly gene [16]. One particular advantage of the fly model is that heart-specific genetic manipulations of critical factors of heart development still allow its survival into adulthood and permit functional analysis, while the same approach in higher model organisms often causes embryonic lethality. In a reverse-genetic screen for genes affecting adult heart function under stress conditions, several members of the CCR4-Not complex were identified to cause dilated cardiomyopathy in the fly, a function that is conserved in a murine model and with implications for human heart disease [17]. One recent example is the role of the YAK/Hippo pathway in mammalian cardiovascular development [18]. Originally identified in Drosophila, YAK/Hippo plays a major role in tissue size control, and recent data show that this function seems to be conserved in mammals. Therefore, lessons learned from *Drosophila* can be (re-)applied to improve our understanding of human heart development and might even be helpful in identifying strategies for preventing and repairing cardiac dysfunction. Here, we review recent advances in the understanding of the molecular-genetic control of heart morphogenesis in *Drosophila*.

## 2. Genetics of *Drosophila* Heart Formation

### 2.1. Genetic Control of Cardiac Specification

The embryonic *Drosophila* heart is a simple, tubular organ comprised of two different cell types: the cardioblasts (CBs), which differentiate into contractile cardiomyocytes of the “working myocardium” and ostia cells, that form the inflow tracts of the heart; and pericardial cells (PCs), most of which become nephrocyte-like cells involved in ultrafiltration that line the outside of the heart tube [19,20]. Pericardial cells have a dual role of also serving as ROS-sensors for the myocardium [21]. The precursors of both the CBs and PCs are specified in the dorsal mesoderm, under the influence of ectodermal TGFβ and WNT signaling pathways [22]. This “cardiac mesoderm” contains the heart’s founder cells that will divide and give rise to both pure and mixed lineages of CBs and PCs (and somatic muscle cells). At this stage, a number of cell fate decisions are made, leading to the specification of subtypes of CBs and PCs, which is under the control of the Notch signaling pathway and its regulators, such as Sanpodo, Numb and ADAM metalloproteinase-disintegrins [22,23,24,25,26]. The identification of Tinman [27,28], a homeobox transcription factor, led to the identification of its mammalian homologue, Nkx2.5, as the first essential factor for the specification of the cardiogenic region in both flies and vertebrates. The genetics and specification cascade downstream of *tin* function in *Drosophila* has been described in great detail (for a detailed review, see Bodmer and Frasch [15]). Briefly, Tinman and the TBX transcription factor, Dorsocross-1/2/3 [29], induce expression of the GATA4-homologue Pannier in the dorsal mesoderm, where Pannier acts as a permissive factor for CB specification [30]. Later, during development, Tinman is subsequently required for the further specification and differentiation of CBs [31], in part through activation of Mef2 [32]. Other examples of cardiac transcription factors studied in *Drosophila* include HAND [33], Neuromancer1/2-TBX20 [34,35], Tail-up/Islet-1 [36,37] and the COUP transcription factor, Seven-up [38]. This genetic and mutational interrogation of cardiogenesis was recently complemented by ChIP-on-chip studies of whole embryos [39,40], which globally identified the transcriptional activity and regulatory networks controlled by Tinman and several other cardiac transcription factors.

### 2.2. Genetics of Heart Morphogenesis

Once specified, the cardiac precursor cells migrate towards the dorsal midline, where they undergo changes in cell shape and assemble to form a linear heart tube. While the bilateral origin of the heart precursors and their fusion at the midline resembles early vertebrate heart morphogenesis (*i.e.*, heart cone formation; compare [41,42]), the linear arrangement of ipsilateral CBs along the anterior-posterior axis and the two-cell composition of the cardiac lumen appear to be morphologically distinct and more similar to mammalian blood vessels than the mammalian heart (e.g., developing mouse aorta [43]). In *Drosophila*, the CBs migrate and undergo morphogenesis in about five hours, which is experimentally advantageous, as it allows cell behavior to be studied in real time throughout heart formation. The CBs and heart are also very accessible for confocal time-lapse and fluorescence imaging of stained whole-mount embryos, since these cells are localized just below the epidermis, which is a single-cell layer and, thus, semi-transparent.

Once the CBs have migrated to the midline, they establish dorsal contacts with the contralateral counterpart, then undergo cell shape changes and ventrally contact the other CBs, thereby enclosing a central lumen. This occurs in a “zipper-like” fashion that closes from both the anterior and posterior ends. The *Drosophila* heart tube is compartmentalized along the A/P axis. A cardiac outflow tract (OFT) is already formed anteriorly while the bilateral heart cells are closing [44], whereas the posterior region of the heart, the “heart proper” forms a larger heart lumen. The CBs in this section differentiate into contractile cardiomyocytes and pairs of cells that form inflow tracts (ostia cells; see [45]). The “aortic” region of the heart is located between the OFT and heart proper and is characterized by the much smaller diameter of the heart lumen and the lack of ostia cells. The heart itself is physically connected to a number of other cell types that also directly contribute to heart function, specifically the alary muscle cells [46,47], which may anchor the heart tube to the epidermis, and the nephrocyte-like pericardial cells [21,48,49,50]. Extracellular matrix proteins, such as laminins and collagens (including Pericardin and Multiplexin), localize in and around the heart and are required for heart assembly and integrity [51,52,53,54,55]. Signaling proteins, such as Integrins [56], as well as G proteins [57], regulate cardiac ECM and cell adhesion directly or indirectly.

Once such pathways are uncovered in *Drosophila*, homologous pathways can be investigated in higher model organisms. For example, studies on the role of the Slit-Robo signaling pathway in *Drosophila* heart morphogenesis have triggered the analysis of homologous genes in vertebrate model organisms. Early on, it was noticed that the ligand Slit is expressed in fly CBs [58]. Subsequent mutational analysis of *slit* and the genes encoding its receptors *robo* and *robo2* revealed that this pathway is indeed critical for maintaining CB chain integrity during migration [59] and for CB cell shape changes during heart morphogenesis [60,61,62]. In vertebrates, Slit/Robo has been implicated in zebrafish heart tube formation [63], where *slit2* is necessary for the migration of endocardial cells during heart tube formation, and in *robo1/2* and *slit3* mutant mouse models, where the formation of the pericardial and venous return is defective [64]. How the Slit-Robo signaling cascade controls morphogenetic events was first worked out for post-mitotic neurons, as they migrate during *Drosophila* nervous system development: binding of the signaling molecule Slit to its receptor Robo induces recruitment of Son-of-Sevenless, a guanine nucleotide exchange factor (GEF), which, in turn, activates the Rho family of GTPases that control the cytoskeletal changes required for the specific migratory response of the neuronal growth cone [65]. The simple *Drosophila* heart tube is well suited to allow the identification of the factors downstream of Slit-Robo, which, in turn, become candidate genes to be analyzed in higher model organisms. Subsequent analyses of the downstream effectors of Slit-Robo using *Drosophila* will likely reveal additional candidate genes to be tested in these models. For example, the *Drosophila* gene encoding Multiplexin (Mp, a collagen15/18) was recently identified to modify Slit-Robo signaling and potentiating Slit activity, resulting in reduced formation of F-actin and altered heart lumen size [54].

### 2.3. Analyzing the Actomyosin Network during Drosophila Heart Morphogenesis

Underneath the instructive layer of cardiac transcription factors and signaling molecules are those proteins that transduce these cues into cellular behavior. For example, CB-specific mutation of *tinman* causes misspecification of cells and morphogenetic defects that are a combination of cell fate changes and inappropriate cell behavior [31].

Elucidating how the cells interpret these internal and external cues is fundamental not only for a comprehensive understanding of organogenesis, but it also offers additional, alternate and specific therapeutic avenues by targeting the effector molecules of disease genes. These basic aspects of cell biology are usually studied in cell culture, where high-resolution analysis of cellular effectors is possible. However, cell culture lacks the complexity of differentiated, three-dimensional multicellular environments, which are present in a living embryo. Therefore, the simplicity of the developing *Drosophila* heart is advantageous to analyze cardiac cell biology during heart morphogenesis and to eventually explain how signaling pathways, such as Slit/Robo and Integrin signaling, give rise to specific cardiac phenotypes when mutated.

Members of the small Rho GTPase family are common molecular hubs that control a large variety of cellular responses, such as cell polarization or cytoskeletal organization (for a review, see [66,67]). Interestingly, the small GTPase *Cdc42* was previously identified as a genetic interactor with *tinman* in adult hearts of *Drosophila* and mice [68]. This interaction was also manifest in the *Drosophila* embryo during heart morphogenesis [69], indicating that Tinman might control heart morphogenesis through Cdc42. During heart development, Cdc42 is necessary for Tin-positive cardioblasts to complete dorsal migration [70], and it controls CB cell shape changes during heart lumen formation. Interestingly, both processes appear to be specific for the small Rho-GTPase Cdc42, but not for the other members, such as Rho1 or Rac1 [69].

**Figure 1:**
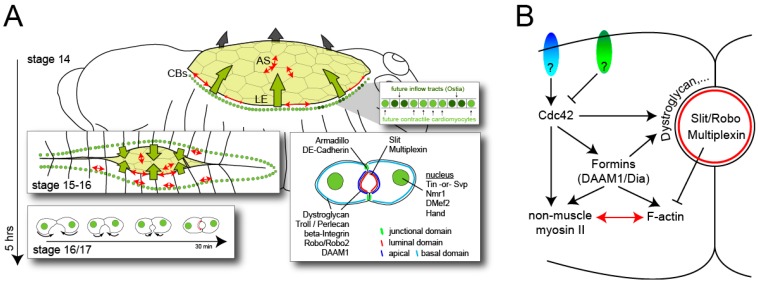
(**a**) Overview of Drosophila heart morphogenesis. Cardioblasts (CBs, green circles) collectively migrate towards the dorsal midline of the embryo. These differentiate later into contractile cardiomyocytes (light green) and ostia cells (dark green). Actomyosin activity can be detected at the edge of the cells of the amnioserosa (AS), at the leading edge of the epidermis (LE) and at the apical side of CBs (red arrows indicate the axis of actomyosin bundles). CB migration is completed within 5 h. Epithelial closure precedes heart closure, and once the CBs make first contact at Stage 16, within 30 min, they actively enclose a central lumen. At that stage, several compartments within the CBs can be identified using several different markers. (**b**) Model showing genetic interactions between Cdc42, formins, actomyosin and the luminal proteins, Slit and Multiplexin. The upstream regulators of Cdc42 (both positive and negative) are yet to be identified.

Lumen formation in *Drosophila* occurs by enclosing of a central lumen, where the CBs actively bend around the luminal space (see Figure 1). Such changes in cell shape are achieved by the activity of the actomyosin network, which has not been studied in detail in the context of *Drosophila* heart formation. Throughout dorsal closure, actomyosin is active in epidermal cells (as “purse-string” actin-myosin cables that pull epidermal sheets towards the midline) and in the cells of the amnioserosa, an extra-embryonic layer that degenerates at late stages of development and, thereby, creates tension by reducing surface area.

Analysis of the expression pattern of the *Drosophila* non-muscle myosin-II *Zipper* (*Zip*) revealed a specific and very dynamic pattern throughout heart morphogenesis [69]. During Stage 15–16, before dorsal closure, Zip repetitively accumulates at the apical side of the CBs, which may be important for apical constriction of CBs to support dorsal closure. During lumen formation, Zip temporarily accumulates at the luminal surface to maintain CB/lumen curvature. In CBs, non-muscle myosin II dynamics is positively regulated by Cdc42 and formin activity, with *Cdc42* also showing genetic interaction with *zip* and the formin, d*DAAM1* [69]. Interestingly, cardiac laterality during zebrafish heart morphogenesis depends on the control of non-muscle myosin-II by BMP signaling [71]. This highlights the need for a better understanding of how the actomyosin network functions *in vivo* and how it is spatio-temporally regulated. 

While these aspects of actomyosin regulation might relate to how force generation shapes the heart, Cdc42/formins also control the heart lumen in a more general fashion. The activation of Cdc42 and formins is sufficient to relocate the entire set of known lumen markers, such as Dystroglycan (Dg), Perlecan/Trol, Slit and Multiplexin, to newly formed, ectopic heart lumina. The latter proteins are part of the extracellular matrix (including anchoring proteins, like Dg, or signaling molecules, like Slit; for an overview, see [72]), and they carry out specific functions during heart morphogenesis and lumen formation, including the downregulation of F-actin itself [54]. In addition, Cdc42 controls the migration of Tin-positive cells before dorsal closure and independent of the known Cdc42 downstream effector p21-activated kinase. The number and hierarchy of genes involved in heart morphogenesis that mediate this cellular behavior are still unknown. In addition, the upstream signals controlling CB migration and lumen formation have yet to be identified. While different signaling pathways (Slit-Robo, Netrin-Unc5, Integrins) are required for heart formation, the molecular-genetic pathways mediating the cellular responses have not been found. In fact, neither Slit-Robo, nor Netrin-Unc5 mutants show changes in non-muscle myosin dynamics [69], which indicates that other pathways are involved, as well. Furthermore, the interaction between CBs and the surrounding tissues (amnioserosa, pericardial cells, dorsal ectoderm) is likely to be critical for heart morphogenesis, as well, but to date, very little is known about how these tissues interact. Recent work showed that regulation of lipid phosphates, Wunen and Wunen2, expressed in the developing heart, is necessary for heart morphogenesis [73], as well as for the timing of dorsal closure, which is delayed in *wun*/*wun2* mutants.

With the advent of affordable whole-genome sequencing, forward genetic screens (such as EMS screens, e.g., in [74]) to identify new players in heart development or genetic interactors of known cardiac factors become more feasible again. In addition, improved RNAi-based approaches, as well as targeted mutations using CRISPR/Cas9 [75,76,77] will allow one to model candidate genes identified from human patients in the fly model and to subsequently functionally evaluate these variants and test for genetic interaction. CRISPR/Cas9 also permits rapid analysis of single genes in zebrafish and mouse models (e.g., see [78]), and these model organisms have a much smaller evolutionary distance to humans compared to flies. Genetic variants that might be responsible for CHD might not be present in the fly genome and, therefore, are a limitation to the analysis of potentially disease-causing alleles. However, the study of genetic interaction (*i.e.*, with core cardiac factors) is still a domain of the fruit fly due to the lack of genetic redundancy for the vast majority of *Drosophila* genes compared to vertebrates, which (in addition to many other advantages; see [79]) makes the fruit fly a promising model for CHD research.

## 3. Conclusions

*Drosophila* is a powerful tool with which to study the genetic and cellular mechanisms of heart formation. Significant genetic homology between flies and humans means that many of the signaling pathways and gene networks uncovered in the fruit fly are relevant in human patients (for an overview see Table 1). The reverse is likely to also be true. Therefore, the fly heart model can be used to screen through candidate CHD-causing genes.

The interactions of Cdc42 with downstream effectors, such as formins, as well as feedback from ECM and signaling pathways therefore represents an experimental entry point to study the cellular events leading to organ formation. Dysregulation of these pathways is likely to contribute to CHD, while understanding the cellular mechanism of heart morphogenesis is necessary for the repair and the engineering of cardiac tissue [80].

**Table 1:** List of selected *Drosophila* genes regulating heart development.

*Drosophila* Gene	Class	Selected Ref.	Vertebrate Ortholog(s)	Selected Ref.
*Diaphanous*	Formin	[69]	DRF3	[10]
*Zipper*	Non-muscle myosin	[69]	NMHC-II A/B/C	[43,81]
*Netrin*	Signaling molecule	[82,83]	NET1/3	[84]
*Slit*	Signaling molecule	[59,60,61,62,69,85]	SLIT1/2/3	[64,86]
*Decapentaplegic*	Signaling molecule	[87]	BMPs	[88]
*Wingless*	Signaling molecule	[89]	WNTs	[90]
*Frazzled*	Signaling Receptor	[83]	NEO1	[91]
*heartless*	Signaling Receptor	[92]	FGFR3/4	[93]
*Notch*	Signaling Receptor	[25]	NOTCH1/2/3	[94]
*Robo1/2/3*	Signaling Receptor	[59,61,62,85]	ROBO1/2/3/4	[64]
*Unc-5*	Signaling Receptor	[82]	UNC5A/B/C/D	[95]
*Kuzbanian*	ADAM10 metalloprotease	[23]	ADAM10	[96]
*Cdc42*	Small Rho GTPase	[69,70]	CDC42	[68,97]
*Multiplexin*	Collagen type XV/XVIII	[54]	COL15/18	[98,99]
*dHand*	Transcription factor	[33,100,101]	HAND1/2	[102,103]
*DMef2*	Transcription factor	[32,104,105]	MEF2A/C/D	[106,107]
*Dorsocross1/2/3*	Transcription factor	[29]	TBX2/3/5	[108,109,110]
*Ladybird-early/late*	Transcription factor	[111,112]	Lbx1/2/3	[113]
*Neuromancer1/2*	Transcription factor	[34,35,114]	TBX20	[115,116]
*Pannier*	Transcription factor	[30,117,118]	GATA4/6	[119]
*Seven-up*	Transcription factor	[38,45]	COUP-TF II	[120]
*Tail-up*	Transcription factor	[36,37,121]	ISL1	[122]
*Tinman*	Transcription factor	[27,28]	NKX2.5	[123]

## References

[1] Marelli A.J., Ionescu-Ittu R., Mackie A.S., Guo L., Dendukuri N., Kaouache M. (2014). Lifetime prevalence of congenital heart disease in the general population from 2000 to 2010. Circulation.

[2] Reller M.D., Strickland M.J., Riehle-Colarusso T., Mahle W.T., Correa A. (2008). Prevalence of congenital heart defects in metropolitan atlanta, 1998–2005. J. Pediatri..

[3] Pierpont M.E., Basson C.T., Benson D.W., Gelb B.D., Giglia T.M., Goldmuntz E., McGee G., Sable C.A., Srivastava D., Webb C.L. (2007). Genetic basis for congenital heart defects: Current knowledge: A scientific statement from the American heart association congenital cardiac defects committee, council on cardiovascular disease in the young: Endorsed by the american academy of pediatrics. Circulation.

[4] Fahed A.C., Gelb B.D., Seidman J.G., Seidman C.E. (2013). Genetics of congenital heart disease: The glass half empty. Circ. Res..

[5] Staudt D., Stainier D. (2012). Uncovering the molecular and cellular mechanisms of heart development using the zebrafish. Ann. Rev. Genet..

[6] Bruneau B.G. (2012). Heart Development.

[7] Bruneau B.G. (2008). The developmental genetics of congenital heart disease. Nature.

[8] Kruithof B.P.T., Duim S.N., Moerkamp A.T., Goumans M.-J. (2012). Tgfβ and BMP signaling in cardiac cushion formation: Lessons from mice and chicken. Differentiation.

[9] Alexander J.M., Bruneau B.G. (2010). Lessons for cardiac regeneration and repair through development. Trends Mol. Med..

[10] Christiaen L., Davidson B., Kawashima T., Powell W., Nolla H., Vranizan K., Levine M. (2008). The transcription/migration interface in heart precursors of ciona intestinalis. Science.

[11] Vogler G., Bodmer R., Akasaka T. (2009). A Drosophila model for congenital heart disease. Drug Discov. Today Dis. Models.

[12] Ocorr K., Vogler G., Bodmer R. (2014). Methods to assess Drosophila heart development, function and aging. Methods.

[13] Reim I., Frasch M. (2009). Genetic and genomic dissection of cardiogenesis in the Drosophila model. Pediatr. Cardiol..

[14] Medioni C., Sénatore S., Salmand P.-A., Lalevée N., Perrin L., Sémériva M. (2009). The fabulous destiny of the Drosophila heart. Curr. Opin. Genet. Dev..

[15] Bodmer R., Frasch M., Harvey R.P., Rosenthal N. (2010). Development and aging of the Drosophila hear. Heart Development and Regeneration.

[16] Mohr S.E., Hu Y., Kim K., Housden B.E., Perrimon N. (2014). Resources for functional genomics studies in Drosophila melanogaster. Genetics.

[17] Neely G.G., Kuba K., Cammarato A., Isobe K., Amann S., Zhang L., Murata M., Elmen L., Gupta V., Arora S. (2010). A global *in vivo* Drosophila RNAi screen identifies Not3 as a conserved regulator of heart function. Cell.

[18] Zhou J. (2014). An emerging role for hippo-yap signaling in cardiovascular development. J. Biomed. Res..

[19] Rizki T.M., Ashburner M., Wright T.R. (1978). The Circulatory System and Associated Cells and Tissues.

[20] Zhang F., Zhao Y., Chao Y., Muir K., Han Z. (2013). Cubilin and amnionless mediate protein reabsorption in Drosophila nephrocytes. J. Am. Soc. Nephrol..

[21] Lim H.-Y., Wang W., Chen J., Ocorr K., Bodmer R. (2014). Ros regulate cardiac function via a distinct paracrine mechanism. Cell Rep..

[22] Bryantsev A.L., Cripps R.M. (2009). Cardiac gene regulatory networks in Drosophila. Biochim. Biophys. Acta.

[23] Albrecht S., Wang S., Holz A., Bergter A., Paululat A. (2006). The adam metalloprotease kuzbanian is crucial for proper heart formation in Drosophila melanogaster. Mech. Dev..

[24] Ward E.J., Skeath J.B. (2000). Characterization of a novel subset of cardiac cells and their progenitors in the Drosophila embryo. Development.

[25] Han Z., Bodmer R. (2003). Myogenic cells fates are antagonized by notch only in asymmetric lineages of the Drosophila heart, with or without cell division. Development.

[26] Park M., Yaich L.E., Bodmer R. (1998). Mesodermal cell fate decisions in Drosophila are under the control of the lineage genes numb, Notch, and sanpodo. Mech. Dev..

[27] Bodmer R. (1993). The gene Tinman is required for specification of the heart and visceral muscles in Drosophila. Development.

[28] Azpiazu N., Frasch M. (1993). Tinman and bagpipe: Two homeo box genes that determine cell fates in the dorsal mesoderm of Drosophila. Genes Dev..

[29] Reim I., Frasch M. (2005). The dorsocross T-box genes are key components of the regulatory network controlling early cardiogenesis in Drosophila. Development.

[30] Klinedinst S.L., Bodmer R. (2003). GATA factor pannier is required to establish competence for heart progenitor formation. Development.

[31] Zaffran S., Reim I., Qian L., Lo P.C., Bodmer R., Frasch M. (2006). Cardioblast-intrinsic Tinman activity controls proper diversification and differentiation of myocardial cells in Drosophila. Development.

[32] Gajewski K., Kim Y., Lee Y.M., Olson E.N., Schulz R.A. (1997). D-MEF2 is a target for Tinman activation during Drosophila heart development. EMBO J..

[33] Han Z., Yi P., Li X., Olson E.N. (2006). Hand, an evolutionarily conserved bHLH transcription factor required for Drosophila cardiogenesis and hematopoiesis. Development.

[34] Miskolczi-McCallum C.M., Scavetta R.J., Svendsen P.C., Soanes K.H., Brook W.J. (2005). The Drosophila melanogaster T-box genes midline and H15 are conserved regulators of heart development. Dev. Biol..

[35] Qian L., Liu J., Bodmer R. (2005). Neuromancer TBX20-related genes (H15/midline) promote cell fate specification and morphogenesis of the Drosophila heart. Dev. Biol..

[36] Tao Y., Wang J., Tokusumi T., Gajewski K., Schulz R.A. (2007). Requirement of the LIM homeodomain transcription factor tailup for normal heart and hematopoietic organ formation in Drosophila melanogaster. Mol. Cell. Biol..

[37] Mann T., Bodmer R., Pandur P. (2009). The Drosophila homolog of vertebrate Islet1 is a key component in early cardiogenesis. Development.

[38] Lo P.C.H., Frasch M. (2001). A role for the coup-tf-related gene seven-up in the diversification of cardioblast identities in the dorsal vessel of Drosophila. Mech. Dev..

[39] Junion G., Spivakov M., Girardot C., Braun M., Gustafson E.H., Birney E., Furlong E.E.M. (2012). A transcription factor collective defines cardiac cell fate and reflects lineage history. Cell.

[40] Jin H., Stojnic R., Adryan B., Ozdemir A., Stathopoulos A., Frasch M. (2013). Genome-wide screens for *in vivo* tinman binding sites identify cardiac enhancers with diverse functional architectures. PLOS Genet..

[41] Rugendorff A., Younossi-Hartenstein A., Hartenstein V. (1994). Embryonic origin and differentiation of the Drosophila heart. Roux’s Arch. Dev. Biol..

[42] Holtzman N.G., Schoenebeck J.J., Tsai H.J., Yelon D. (2007). Endocardium is necessary for cardiomyocyte movement during heart tube assembly. Development.

[43] Strilić B., Kucera T., Eglinger J., Hughes M.R., McNagny K.M., Tsukita S., Dejana E., Ferrara N., Lammert E. (2009). The molecular basis of vascular lumen formation in the developing mouse aorta. Dev. Cell.

[44] Zikova M., da Ponte J.-P., Dastugue B., Jagla K. (2003). Patterning of the cardiac outflow region in Drosophila. Proc. Natl. Acad. Sci. USA.

[45] Molina M.R., Cripps R.M. (2001). Ostia, the inflow tracts of the Drosophila heart, develop from a genetically distinct subset of cardial cells. Mech. Dev..

[46] LaBeau E.M., Trujillo D.L., Cripps R.M. (2009). Bithorax complex genes control alary muscle patterning along the cardiac tube of Drosophila. Mech. Dev..

[47] Boukhatmi H., Schaub C., Bataillé L., Reim I., Frendo J.-L., Frasch M., Vincent A. (2014). An Org-1-Tup transcriptional cascade reveals different types of alary muscles connecting internal organs in Drosophila. Development.

[48] Zhuang S., Shao H., Guo F., Trimble R., Pearce E., Abmayr S.M. (2009). Sns and Kirre, the Drosophila orthologs of Nephrin and Neph1, direct adhesion, fusion and formation of a slit diaphragm-like structure in insect nephrocytes. Development.

[49] Weavers H., Prieto-Sánchez S., Grawe F., Garcia-López A., Artero R., Wilsch-Bräuninger M., Ruiz-Gómez M., Skaer H., Denholm B. (2009). The insect nephrocyte is a podocyte-like cell with a filtration slit diaphragm. Nature.

[50] Buechling T., Akasaka T., Vogler G., Ruiz-Lozano P., Ocorr K.A., Bodmer R. (2009). Non-autonomous modulation of heart rhythm, contractility and morphology in adult fruit flies. Dev. Biol..

[51] Hollfelder D., Frasch M., Reim I. (2014). Distinct functions of the laminin β LN domain and collagen IV during cardiac extracellular matrix formation and stabilization of alary muscle attachments revealed by ems mutagenesis in Drosophila. BMC Dev. Biol..

[52] Yarnitzky T., Volk T. (1995). Laminin is required for heart, somatic muscles, and gut development in the Drosophila embryo. Dev. Biol..

[53] Chartier A., Zaffran S., Astier M., Sémériva M., Gratecos D. (2002). Pericardin, a Drosophila type IV collagen-like protein is involved in the morphogenesis and maintenance of the heart epithelium during dorsal ectoderm closure. Development.

[54] Harpaz N., Ordan E., Ocorr K., Bodmer R., Volk T. (2013). Multiplexin promotes heart but not aorta morphogenesis by polarized enhancement of Slit/Robo activity at the heart lumen. PLOS Genet..

[55] Volk T., Wang S., Rotstein B., Paululat A. (2014). Matricellular proteins in development: Perspectives from the Drosophila heart. Matrix Biol..

[56] Vanderploeg J., Vazquez Paz L.L., MacMullin A., Jacobs J.R. (2012). Integrins are required for cardioblast polarisation in Drosophila. BMC Dev. Biol..

[57] Yi P., Johnson A.N., Han Z., Wu J., Olson E.N. (2008). Heterotrimeric g proteins regulate a noncanonical function of septate junction proteins to maintain cardiac integrity in Drosophila. Dev. Cell.

[58] Rothberg J.M., Jacobs J.R., Goodman C.S., Artavanis-Tsakonas S. (1990). Slit: An extracellular protein necessary for development of midline glia and commissural axon pathways contains both EGF and LRR domains. Genes Dev..

[59] Qian L., Liu J., Bodmer R. (2005). Slit and Robo control cardiac cell polarity and morphogenesis. Curr. Biol..

[60] Medioni C., Astier M., Zmojdzian M., Jagla K., Semeriva M. (2008). Genetic control of cell morphogenesis during Drosophila melanogaster cardiac tube formation. J. Cell Biol..

[61] MacMullin A., Jacobs J.R. (2006). Slit coordinates cardiac morphogenesis in Drosophila. Dev. Biol..

[62] Santiago-Martínez E., Soplop N.H., Patel R., Kramer S.G. (2008). Repulsion by Slit and roundabout prevents Shotgun/E-cadherin-mediated cell adhesion during Drosophila heart tube lumen formation. J. Cell Biol..

[63] Fish J.E., Wythe J.D., Xiao T., Bruneau B.G., Stainier D.Y.R., Srivastava D., Woo S. (2011). A Slit/miR-218/Robo regulatory loop is required during heart tube formation in zebrafish. Development.

[64] Mommersteeg M.T.M., Andrews W.D., Ypsilanti A.R., Zelina P., Yeh M.L., Norden J., Kispert A., Chédotal A., Christoffels V.M., Parnavelas J.G. (2012). Slit-Robo signaling regulates the development of the cardiac systemic venous return and pericardium. Circ. Res..

[65] Yang L., Bashaw G.J. (2006). Son of sevenless directly links the Robo receptor to Rac activation to control axon repulsion at the midline. Neuron.

[66] Hanna S., El-Sibai M. (2013). Signaling networks of Rho GTPases in cell motility. Cell. Signal..

[67] Iden S., Collard J.G. (2008). Crosstalk between small GTPases and polarity proteins in cell polarization. Nat. Rev. Mol. Cell Biol..

[68] Qian L., Wythe J.D., Liu J., Cartry J., Vogler G., Mohapatra B., Otway R.T., Huang Y., King I.N., Maillet M. (2011). Tinman/Nkx2–5 acts via miR-1 and upstream of Cdc42 to regulate heart function across species. J. Cell Biol..

[69] Vogler G., Liu J., Iafe T.W., Migh E., Mihály J., Bodmer R. (2014). Cdc42 and formin activity control non-muscle myosin dynamics during Drosophila heart morphogenesis. J. Cell Biol..

[70] Swope D., Kramer J., King T.R., Cheng Y.-S., Kramer S.G. (2014). Cdc42 is required in a genetically distinct subset of cardiac cells during Drosophila dorsal vessel closure. Dev. Biol..

[71] Veerkamp J., Rudolph F., Cseresnyes Z., Priller F., Otten C., Renz M., Schaefer L., Abdelilah-Seyfried S. (2013). Unilateral dampening of BMP activity by nodal generates cardiac left-right asymmetry. Dev. Cell.

[72] Brandt R., Paululat A. (2013). Microcompartments in the Drosophila heart and the mammalian brain: General features and common principles. Biol. Chem..

[73] Haack T., Schneider M., Schwendele B., Renault A.D. (2014). Drosophila heart cell movement to the midline occurs through both cell autonomous migration and dorsal closure. Dev. Biol..

[74] Reim I., Hollfelder D., Ismat A., Frasch M. (2012). The FGF8-related signals Pyramus and Thisbe promote pathfinding, substrate adhesion, and survival of migrating longitudinal gut muscle founder cells. Dev. Biol..

[75] Harrison M.M., Jenkins B.V., O’Connor-Giles K.M., Wildonger J. (2014). A crispr view of development. Genes Dev..

[76] Mohr S.E., Smith J.A., Shamu C.E., Neumüller R.A., Perrimon N. (2014). RNAi screening comes of age: Improved techniques and complementary approaches. Nat. Rev. Mol. Cell Biol..

[77] Gratz S.J., Cummings A.M., Nguyen J.N., Hamm D.C., Donohue L.K., Harrison M.M., Wildonger J., O’Connor-Giles K.M. (2013). Genome engineering of Drosophila with the CRISPR RNA-guided Cas9 nuclease. Genetics.

[78] Long C., McAnally J.R., Shelton J.M., Mireault A.A., Bassel-Duby R., Olson E.N. (2014). Prevention of muscular dystrophy in mice by CRISPR/Cas9-mediated editing of germline DNA. Science.

[79] Bier E. (2005). Drosophila, the golden bug, emerges as a tool for human genetics. Nat. Rev. Genet..

[80] Xin M., Olson E.N., Bassel-Duby R. (2013). Mending broken hearts: Cardiac development as a basis for adult heart regeneration and repair. Nat. Rev. Mol. Cell Biol..

[81] Tullio A.N., Accili D., Ferrans V.J., Yu Z.X., Takeda K., Grinberg A., Westphal H., Preston Y.A., Adelstein R.S. (1997). Nonmuscle myosin II-B is required for normal development of the mouse heart. Proc. Natl. Acad. Sci. USA.

[82] Albrecht S., Altenhein B., Paululat A. (2011). The transmembrane receptor uncoordinated5 (UNC5) is essential for heart lumen formation in Drosophila melanogaster. Dev. Biol..

[83] Macabenta F.D., Jensen A.G., Cheng Y.-S., Kramer J.J., Kramer S.G. (2013). Frazzled/DCC facilitates cardiac cell outgrowth and attachment during Drosophila dorsal vessel formation. Dev.Biol..

[84] Lai Wing Sun K., Correia J.P., Kennedy T.E. (2011). Netrins: Versatile extracellular cues with diverse functions. Development.

[85] Santiago-Martínez E., Soplop N.H., Kramer S.G. (2006). Lateral positioning at the dorsal midline: Slit and roundabout receptors guide Drosophila heart cell migration. Proc. Natl. Acad. Sci. USA.

[86] Medioni C., Bertrand N., Mesbah K., Hudry B., Dupays L., Wolstein O., Washkowitz A.J., Papaioannou V.E., Mohun T.J., Harvey R.P. (2010). Expression of Slit and Robo genes in the developing mouse heart. Dev. Dyn..

[87] Frasch M. (1995). Induction of visceral and cardiac mesoderm by ectodermal Dpp in the early Drosophila embryo. Nature.

[88] Rojas A., de Val S., Heidt A.B., Xu S.-M., Bristow J., Black B.L. (2005). GATA4 expression in lateral mesoderm is downstream of BMP4 and is activated directly by Forkhead and GATA transcription factors through a distal enhancer element. Development.

[89] Park M., Wu X., Golden K., Axelrod J.D., Bodmer R. (1996). The wingless signaling pathway is directly involved in Drosophila heart development. Dev. Biol..

[90] Ueno S., Weidinger G., Osugi T., Kohn A.D., Golob J.L., Pabon L., Reinecke H., Moon R.T., Murry C.E. (2007). Biphasic role for WNT/beta-catenin signaling in cardiac specification in zebrafish and embryonic stem cells. Proc. Natl. Acad. Sci. USA.

[91] Wilson N.H., Key B. (2007). Neogenin: One receptor, many functions. Int. J. Biochem. Cell Biol..

[92] Wilson R., Vogelsang E., Leptin M. (2005). FGF signalling and the mechanism of mesoderm spreading in Drosophila embryos. Development.

[93] Marguerie A., Bajolle F., Zaffran S., Brown N.A., Dickson C., Buckingham M.E., Kelly R.G. (2006). Congenital heart defects in Fgfr2-IIIb and Fgf10 mutant mice. Cardiovasc. Res..

[94] Niessen K., Karsan A. (2008). Notch signaling in cardiac development. Circ. Res..

[95] Navankasattusas S., Whitehead K.J., Suli A., Sorensen L.K., Lim A.H., Zhao J., Park K.W., Wythe J.D., Thomas K.R., Chien C.-B. (2008). The netrin receptor UNC5B promotes angiogenesis in specific vascular beds. Development.

[96] Hartmann D., de Strooper B., Serneels L., Craessaerts K., Herreman A., Annaert W., Umans L., Lubke T., Lena Illert A., von Figura K. (2002). The disintegrin/metalloprotease ADAM 10 is essential for notch signalling but not for alpha-secretase activity in fibroblasts. Hum. Mol. Genet..

[97] Maillet M., Lynch J.M., Sanna B., York A.J., Zheng Y., Molkentin J.D. (2009). Cdc42 is an antihypertrophic molecular switch in the mouse heart. J. Clin. Investig..

[98] Rasi K., Piuhola J., Czabanka M., Sormunen R., Ilves M., Leskinen H., Rysä J., Kerkelä R., Janmey P., Heljasvaara R. (2010). Collagen XV is necessary for modeling of the extracellular matrix and its deficiency predisposes to cardiomyopathy. Circ. Res..

[99] Eklund L., Piuhola J., Komulainen J., Sormunen R., Ongvarrasopone C., Fássler R., Muona A., Ilves M., Ruskoaho H., Takala T.E. (2001). Lack of type XV collagen causes a skeletal myopathy and cardiovascular defects in mice. Proc. Natl. Acad. Sci. USA.

[100] Lo P.C.H., Zaffran S., Sénatore S., Frasch M. (2007). The Drosophila hand gene is required for remodeling of the developing adult heart and midgut during metamorphosis. Dev. Biol..

[101] Han Z., Olson E.N. (2005). Hand is a direct target of tinman and GATA factors during Drosophila cardiogenesis and hematopoiesis. Development.

[102] Firulli A.B., McFadden D.G., Lin Q., Srivastava D., Olson E.N. (1998). Heart and extra-embryonic mesodermal defects in mouse embryos lacking the bHLH transcription factor hand1. Nat. Genet..

[103] Cheng Z., Lib L., Li Z., Liu M., Yan J., Wang B., Ma X. (2012). Two novel hand1 mutations in chinese patients with ventricular septal defect. Clin. Chim. Acta.

[104] Cripps R.M., Lovato T.L., Olson E.N. (2004). Positive autoregulation of the myocyte enhancer factor-2 myogenic control gene during somatic muscle development in Drosophila. Dev. Biol..

[105] Lilly B., Zhao B., Ranganayakulu G., Paterson B.M., Schulz R.A., Olson E.N. (1995). Requirement of MADS domain transcription factor D-MEF2 for muscle formation in Drosophila. Science.

[106] Lin Q., Schwarz J., Bucana C., Olson E.N. (1997). Control of mouse cardiac morphogenesis and myogenesis by transcription factor mef2c. Science.

[107] Naya F.J., Black B.L., Wu H., Bassel-Duby R., Richardson J.A., Hill J.A., Olson E.N. (2002). Mitochondrial deficiency and cardiac sudden death in mice lacking the MEF2A transcription factor. Nat. Med..

[108] Bruneau B.G., Nemer G., Schmitt J.P., Charron F., Robitaille L., Caron S., Conner D.A., Gessler M., Nemer M., Seidman C.E. (2001). A murine model of holt-oram syndrome defines roles of the T-box transcription factor TBX5 in cardiogenesis and disease. Cell.

[109] Harrelson Z., Kelly R.G., Goldin S.N., Gibson-Brown J.J., Bollag R.J., Silver L.M., Papaioannou V.E. (2004). TBX2 is essential for patterning the atrioventricular canal and for morphogenesis of the outflow tract during heart development. Development.

[110] Bakker M.L., Boukens B.J., Mommersteeg M.T.M., Brons J.F., Wakker V., Moorman A.F.M., Christoffels V.M. (2008). Transcription factor TBX3 is required for the specification of the atrioventricular conduction system. Circ. Res..

[111] Junion G., Bataillé L., Jagla T., Da Ponte J.P., Tapin R., Jagla K. (2007). Genome-wide view of cell fate specification: Ladybird acts at multiple levels during diversification of muscle and heart precursors. Genes Dev..

[112] Jagla K., Jagla T., Heitzler P., Dretzen G., Bellard F., Bellard M. (1997). Ladybird, a tandem of homeobox genes that maintain late wingless expression in terminal and dorsal epidermis of the Drosophila embryo. Development.

[113] Schäfer K., Neuhaus P., Kruse J., Braun T. (2003). The homeobox gene Lbx1 specifies a subpopulation of cardiac neural crest necessary for normal heart development. Circ. Res..

[114] Reim I., Mohler J.P., Frasch M. (2005). TBX20-related genes, mid and H15, are required for tinman expression, proper patterning, and normal differentiation of cardioblasts in Drosophila. Mech. Dev..

[115] Kirk E.P., Sunde M., Costa M.W., Rankin S.A., Wolstein O., Castro M.L., Butler T.L., Hyun C., Guo G., Otway R. (2007). Mutations in cardiac T-box factor gene TBX20 are associated with diverse cardiac pathologies, including defects of septation and valvulogenesis and cardiomyopathy. Am. J. Hum. Genet..

[116] Hammer S., Toenjes M., Lange M., Fischer J.J., Dunkel I., Mebus S., Grimm C.H., Hetzer R., Berger F., Sperling S. (2008). Characterization of TBX20 in human hearts and its regulation by TFAP2. J. Cell. Biochem..

[117] Alvarez A.D., Shi W., Wilson B.A., Skeath J.B. (2003). Pannier and pointedP2 act sequentially to regulate Drosophila heart development. Development.

[118] Gajewski K., Fossett N., Molkentin J.D., Schulz R.A. (1999). The zinc finger proteins pannier and GATA4 function as cardiogenic factors in Drosophila. Development.

[119] Zhou P., He A., Pu W.T. (2012). Regulation of GATA4 transcriptional activity. Cardiovascular Development and Disease.

[120] Pereira F.A., Qiu Y., Zhou G., Tsai M.J., Tsai S.Y. (1999). The orphan nuclear receptor COUP-TFII is required for angiogenesis and heart development. Genes Dev..

[121] Zmojdzian M., Jagla K. (2013). Tailup plays multiple roles during cardiac outflow assembly in Drosophila. Cell Tissue Res..

[122] Cai C.-L., Liang X., Shi Y., Chu P.-H., Pfaff S.L., Chen J., Evans S. (2003). Isl1 identifies a cardiac progenitor population that proliferates prior to differentiation and contributes a majority of cells to the heart. Dev. Cell.

[123] Akazawa H., Komuro I. (2005). Cardiac transcription factor Csx/Nkx2-5: Its role in cardiac development and diseases. Pharmacol. Ther..

